# Insights into *Aspergillus fumigatus* Colonization in Cystic Fibrosis and Cross-Transmission between Patients and Hospital Environments

**DOI:** 10.3390/jof10070461

**Published:** 2024-06-29

**Authors:** Laís Pontes, Ana Luisa Perini Leme Giordano, Franqueline Reichert-Lima, Caio Augusto Gualtieri Beraquet, Guilherme Leite Pigolli, Teppei Arai, José Dirceu Ribeiro, Aline Cristina Gonçalves, Akira Watanabe, Gustavo Henrique Goldman, Maria Luiza Moretti, Angélica Zaninelli Schreiber

**Affiliations:** 1 Faculdade de Ciências Médicas, Universidade Estadual de Campinas, Campinas 13083-887, Brazil; analuisa.giordano@gmail.com (A.L.P.L.G.); franque.reichert@gmail.com (F.R.-L.); caioberaquet@gmail.com (C.A.G.B.); gpigolli@gmail.com (G.L.P.); jdirceur@unicamp.br (J.D.R.); licgoncalves@yahoo.com.br (A.C.G.); moretti.luiza@gmail.com (M.L.M.); 2 Division of Clinical Research, Medical Mycology Research Center, Chiba University, Chiba 260-0856, Japan; arai.teppei@chiba-u.jp (T.A.); fewata@faculty.chiba-u.jp (A.W.); 3 Faculdade de Ciências Farmacêuticas de Ribeirão Preto, Universidade de São Paulo, Ribeirão Preto 14040-903, Brazil; ggoldman@usp.br

**Keywords:** *Aspergillus fumigatus*, cystic fibrosis, cross-transmission

## Abstract

Background: Approximately 60% of individuals with cystic fibrosis (CF) are affected by *Aspergillus fumigatus* infection. This condition is correlated with a decline in lung function and is identified as an independent risk factor contributing to hospital admissions among CF patients. This study investigates the dynamic interplay of *A. fumigatus* within the context of CF patients, tracing its evolution over time, with a specific emphasis on colonization dynamics. Methods: An analysis was conducted on 83 sequential *A. fumigatus* isolates derived from sputum samples of six patients receiving care at a renowned CF hospital in Brazil. Employing microsatellite genotyping techniques, alongside an investigation into *cyp*51A gene mutations, this research sheds light on the genetic variations, colonization, and resistance of *A. fumigatus* within the CF respiratory environment. Results: Our research findings indicate that CF patients can harbor *A. fumigatus* strains from the same clonal complexes for prolonged periods. Additionally, we identified that clinical isolates have the potential to spread among patients in the same healthcare facility, evidencing hospital contamination. Two patients who underwent long-term Itraconazole treatment did not show phenotypic resistance. However, one of these patients exhibited mutations in the *cyp*51A gene, indicating the need to monitor resistance to azoles in these patients colonized for long periods by *A. fumigatus*. We also observed co-colonization or co-infection involving multiple genotypes in all patients over time. Conclusion: This comprehensive examination offers valuable insights into the pathogenesis of *A. fumigatus* infections in CF patients, potentially shaping future therapeutic strategies and management approaches. This enhanced understanding contributes to our knowledge of *A. fumigatus* impact on disease progression in individuals with cystic fibrosis. Additionally, the study provides evidence of cross-contamination among patients undergoing treatment at the same hospital.

## 1. Introduction

Cystic fibrosis (CF) is a life-limiting, multisystemic, autosomal recessive disease caused by various mutations in the gene responsible for encoding the cystic fibrosis transmembrane conductance regulator (CFTR) gene. The CFTR gene is associated with producing a chloride-conducting transmembrane channel [1,2].

In individuals with CF, a significant portion of morbidity and mortality is attributed to chronic lung disease [3]. The recent integration of CF transmembrane conductance regulator (CFTR) modulators into clinical practice appears to decrease pulmonary exacerbations and slow the rate of decline in lung function. However, it is important to note that these modulators cannot reverse existing lung damage [4]. Despite the introduction of new treatments, pulmonary infections remain a significant prognostic challenge in individuals with CF. While there have been notable advancements in CF research and treatment, the emphasis has predominantly been on bacterial pathogens, with limited attention given to fungal species [5].

Fungal colonization and infections are frequent occurrences in CF patients, and *Aspergillus fumigatus* is commonly isolated in this patient group [6]. Approximately 60% of CF patients are affected by *A. fumigatus*, which is associated with an accelerated decline in lung function [7]. *A. fumigatus* is a widely distributed mold species, and its spores are regularly inhaled [8]. The susceptibility of CF patients to *A. fumigatus*-related lung diseases varies, ranging from persistent infection and bronchitis to allergic reactions and airway-invasive aspergillosis [9,10,11]. While *A. fumigatus* colonization may not always manifest clinically, studies suggest that persistent *A. fumigatus* infection constitutes a significant and independent risk factor for hospital admissions in CF patients [12].

Studies have demonstrated that CF patients may exhibit one or more genotypes of *A. fumigatus* [13,14]. Genotyping can be achieved through microsatellite typing [14,15]. While this technique cannot distinguish between colonization and infection, it can differentiate various isolates from the same patient or related isolates from different patients. It is a crucial tool to determine whether individuals are colonized or infected by microorganisms with the same clonal origin [15,16]. Additionally, specific genotypes may be consistently isolated, indicating continuous colonization [14]. Different genotypes may also exhibit distinct azole susceptibility profiles, and detecting these differences is crucial in managing these patients [17,18].

There has been a growing concern about the impact of *A. fumigatus* on respiratory manifestations and the use of antifungal therapy in CF [17]. The increase in azole-resistant *A. fumigatus* isolation is suggested to be a result of prolonged therapeutic [19] or environmental exposure to azoles [20,21]. The detection of azole-resistant *A. fumigatus* has been a matter of interest for years [22,23]. Resistant *A. fumigatus* isolates have also been associated with treatment failure in individuals with aspergillosis [24]. Most resistant strains harbor a tandem repeat in the promoter region of the *cyp*51A gene, along with point mutations leading to amino acid changes in *cyp*51A [25,26]. Testing for azole resistance is essential for epidemiological analysis and clinical applications due to the potential for treatment failure outcomes [24].

Several molecular techniques have been employed to detect *cyp*51A mutations, including DNA sequencing [27], real-time PCR [28], loop-mediated isothermal amplification (LAMP) [29], and surveyor nuclease assay [30]. Each method possesses its own set of advantages and disadvantages. DNA sequencing is considered the gold standard and precise but can be expensive and labor-intensive, as is the case with real-time PCR and LAMP. In contrast, the surveyor nuclease assay is a simpler and faster screening technique with the potential for broad applications [30].

In this research endeavor, our primary objective was to comprehensively characterize the sequential isolates of *A. fumigatus* obtained from a reference hospital for treating CF in Brazil. This investigation aimed to delve into the intricate dynamics of colonization, a crucial aspect for unraveling the progression of diseases in patients harboring *A. fumigatus* over extended periods and investigate azole susceptibility. To achieve this, we employed microsatellite genotyping and conducted a thorough examination of *cyp*51A gene mutations utilizing the surveyor nuclease assay.

## 2. Materials and Methods

### 2.1. Fungal Isolates

Eighty sequential isolates of *A. fumigatus* were gathered from six CF patients undergoing treatment at the Hospital de Clinicas -UNICAMP - Campinas, São Paulo, Brazil, from 2014 to 2018. These isolates were cultured from sputum samples, and their identification was initially conducted using conventional morphological methods. Following identification, the isolates were preserved in distilled water at room temperature [31]. For this study, all isolates were cultivated on Sabouraud Dextrose Agar (SDA; Difco, Sparks, MD, USA) to facilitate the subsequent experiments.

### 2.2. Microbiological Identification

The micromorphology and macromorphology of the isolates were observed following their growth on SDA and Potato Dextrose Agar (PDA; Difco, Sparks, MD, USA) [32].

### 2.3. Molecular Identification

Genomic DNA extraction was performed after 48–72 h of growth at 25 °C on SDA plates using a DNeasy tissue kit (Qiagen, Valencia, CA, USA) following the manufacturer’s instructions. Comparative DNA analyses of beta-tubulin (β-tubulin 2A/B) sequences were conducted for species confirmation [33]. The obtained nucleotide sequences were analyzed using Geneious^®^ 8.1 (Biomatters Ltd. 2015, Newark, NJ, USA) and compared with external databases.

### 2.4. Microsatellites

For genotyping, PCR was conducted using nine pairs of primers, and approximately 400 base pairs from the isolates were sequenced, following previously established procedures. The repeat numbers for each region (2A, 2B, 2C, 3A, 3B, 3C, 4A, 4B, and 4C) were determined from the DNA sequence [34].

### 2.5. Screening of cyp51A Point Mutations

The strains underwent screening for *cyp*51A point mutations using the Surveyor Nuclease assay following established protocols [30]. In brief, the *cyp*51A amplicons from each strain were hybridized with a reference *cyp*51A amplicon. Subsequently, they were treated with Surveyor Nuclease (Integrated DNA Technologies, Inc., Commercial Park-Coralville, IW, USA), an endonuclease capable of cleaving sites with DNA mismatches and distortions. Both cleaved and uncleaved fragments were then visualized through electrophoresis.

### 2.6. Sequencing of cyp51A Gene

The positive isolates in the surveyor nuclease assay underwent further analysis for mutations in the *cyp*51A gene. DNA extraction was carried out using 48 h fungal cultures following the previously mentioned procedure. Oligonucleotides AF1P1 (F/R), AF2P1 (F/R), AF3P1 (F/R), and AF4P1 (F/R) were employed for the amplification and sequence analysis of *cyp*51A [25,35].

### 2.7. Broth Microdilution Test (BMD)

The Minimum Inhibitory Concentration (MIC) for azole values was determined for the strains that were positive in the surveyor nuclease assay. The MIC was determined visually after incubation at 35 °C for 48 h following the guidelines outlined in the Clinical and Laboratory Standards Institute M38-A3 [36]. Antifungal susceptibility testing was conducted using pre-prepared dry plates (Eiken Chemical Co., Tokyo, Japan) with an inoculum of 2.5 × 10^4^ CFU/mL of conidia.

The evaluated antifungal agents were Itraconazole (ITC), Voriconazole (VRC), and Posaconazole (POS), ranging from 0.015 to 8 µg/mL. POS was not available on the plate and was prepared separately: POS (Sigma-Aldrich, St. Louis, MO, USA) was dissolved in water and then diluted in RPMI 1640 (Sigma-Aldrich).

Quality controls were incorporated into each test, including *Aspergillus flavus* ATCC 204304, *Candida parapsilosis* ATCC 22019, and *Candida krusei* ATCC 6258.

## 3. Results

### 3.1. Isolates Identification and Microsatellite Genotyping

Based on the morphological identification and DNA sequencing of the β-tubulin 2A/B regions, all isolates were confirmed as *A. fumigatus*. The patients’ ages ranged from 21 to 27 years, with 80% being male.

**Figure 1:**
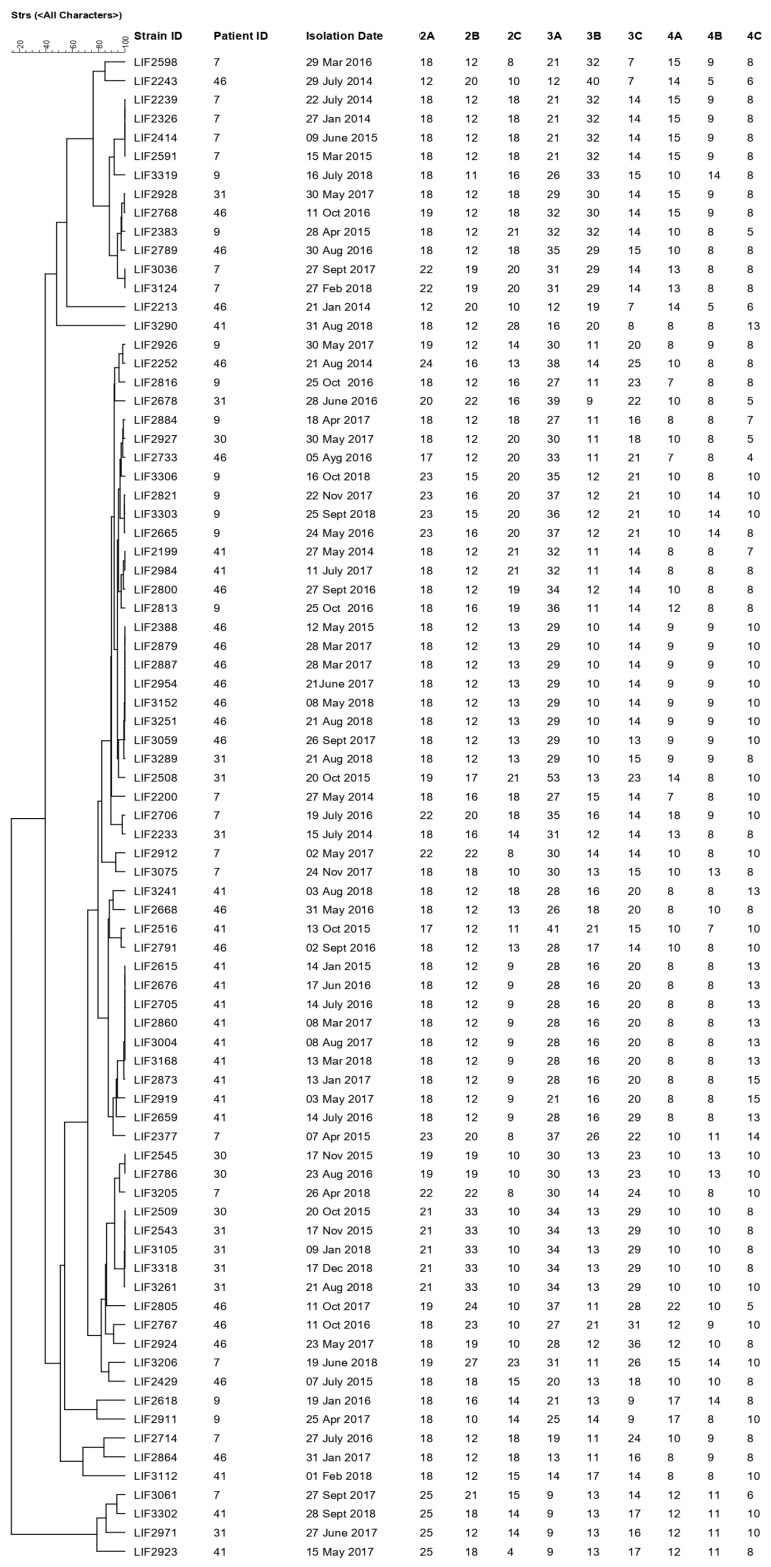
Genotypic relationship between 80 isolates of *Aspergillus fumigatus* isolates. The dendrogram is based on a categorical analysis of nine microsatellite markers in combination with an unweighted pair group method with arithmetic mean (UPGMA) clustering using BioNumerics V7.6 software (Applied Math Inc., Austin, TX, USA).

The genotyping results of nine microsatellites illustrate the genetic relationships among the 80 sequential isolates of *A. fumigatus* obtained from six CF patients (Figure 1). The dendrogram reveals a genetic correlation between the strains from patients 46 and 31 and 30 and 31, with several isolates exhibiting identical microsatellite patterns among the patients (Figure 1).

Patient 7 displayed a genetic correlation among isolates LIF 2239, LIF 2326, LIF 2414, and LIF 2591, isolated from 2014 to 2015 (Figure 1), belonging to the same clonal complex. In contrast, patient 9 showed colonization by different clones of *A. fumigatus* from 2015 to 2018, except for isolates LIF 2821, LIF 3303, and LIF 2665, which belong to the same clonal complex; notably, only LIF 3303 has two ST difference (primers 2B and 3A). Patient 31 exhibited five different clonal complexes, emphasizing colonization by the same strain in 2015 (LIF 2543) and 2018 (LIF 3105, LIF 3318). Patient 41 was colonized from 2015 to 2018 by seven isolates (LIF 2615, LIF 2873, LIF 2676, LIF 2705, LIF 2860, LIF 3004, and LIF 3168) sharing the same clonal origin. Lastly, patient 46 displayed a genotyping correlation among isolates LIF 2388, LIF 2879, LIF 2887, LIF 2954, LIF 3152, LIF 3251, and LIF 3059, isolated over three years.

Patient 46 was treated during two hospital stays with (1) ITC 400 mg/day from September to November 2016, leading to the isolation of clinical isolates LIF 2733, LIF 2767, LIF 2768, LIF 2791, LIF 2800, and LIF 2805; and (2) ITC 200 mg/day from July to September 2017, resulting in the isolation of clinical isolates LIF 2934 and LIF 3059.

### 3.2. Screening and Detection of cyp51A Point Mutations

For the surveyor nuclease assay, only seven isolates were positive (LIF 3061, LIF 2509, LIF 2543, LIF 3105, LIF 3261, LIF 3318, and LIF 3302), which belong to four different patients. These same isolates were subjected to the sequencing of the *cyp*51A gene to identify the amino acid exchange (Table 1).

For patients who were positive on surveyor nuclease, only patient 31 received ITC treatment. Patient 31 underwent two hospitalizations with (1) ITC 200 mg/day for 30 days in October 2015, resulting in the isolation of clinical isolate LIF 2543, and (2) ITC 200 mg/day for 30 days in January 2018, with clinical isolate LIF 3105 isolated during this period.

All seven clinical isolates displayed lower MIC results for VRC, ITC, and POS, indicating no azole resistance (Table 1).

## 4. Discussion

Inflammation and chronic lung infections are primary contributors to morbidity and mortality in CF patients. While diagnostic and treatment efforts have traditionally concentrated on bacterial infections, recent insights into the lung and gut microbiome emphasize the significance of fungal species, particularly *C. albicans* and *A. fumigatus* [9,37]. Despite this, the clinical role of *A. fumigatus* in the CF patient’s lung remains inadequately elucidated, although accumulating evidence suggests its harmful impact on CF lung disease [38].

*Aspergillus fumigatus* exhibits a wide prevalence range of 5 to 54% [39] and is recognized for causing conditions such as allergic bronchopulmonary aspergillosis (ABPA), bronchitis, and pneumonia, often presenting challenges in treatment [40]. The colonization of *A. fumigatus* in a chronic CF patient can stem from various sources: (i) different patients sharing the same strain genotype, likely due to hospital environmental contamination as these patients do not reside together or in the exact locations; (ii) an individual harboring different strain genotypes; and (iii) prolonged colonization by the same strain genotype [13,18,41]. This comprehension is vital as it unveils diverse strategies of fungal colonization and provides insights into the evolution of azole resistance [13,18]. 

We employed microsatellite genotyping to comprehend and distinguish between these various scenarios. Through this technique, we could categorize isolates sharing the same clonal origin and differing by 1 to 2 Sequence Types (STs) as part of a clonal complex. Some isolates outside the complex exhibited differences of more than 2 STs from each other, potentially attributed to the instability of the markers STRAf 3A and STRAf 3C. This factor should be considered when interpreting the results [42].

We have observed distinct patterns among our six patients: (i) patients 46, 31, and 30 shared the same strains, indicating potential hospital contamination; (ii) patients 9 and 30 exhibited strains from three and five different clonal complexes, respectively, suggesting infection by varied environmental strains during colonization; and (iii) patients 7 and 41 demonstrated different strains from the same clonal origin, implying prolonged colonization by the same strain genotype.

Our findings align with previous studies emphasizing the considerable diversity in *A. fumigatus* populations among patients and the variability within isolates from the same patient [13,43]. *A. fumigatus* conidia found in the hospital environment may contribute to an increased and persistent burden for CF patients [44]. Engel et al.’s study [45] conducted at a CF treatment center in the Netherlands identified *A. fumigatus* in aerosols. Among the 15 patients studied, 2 were colonized by the same genotype, illustrating the potential airborne spread of this microorganism indoors. A study proposes that *A. fumigatus* in chronically infected patients follows an evolutionary trajectory leading to microorganisms with specific adaptations better suited to the pulmonary environment [46].

Cystic fibrosis patients, consequently, exhibit pulmonary conditions favorable to *A. fumigatus* colonization and infection [3,46]. *A. fumigatus* that is well-adapted to the lung environment may have a higher likelihood, compared to environmental isolates, of successfully colonizing and persisting in the airways of CF patients [46]. The presence of *A. fumigatus* in the lungs of CF patients might offer opportunities for these fungi to acquire specific characteristics that enhance their ability to thrive in diverse host environments [46]. Therefore, closely monitoring sequential *A. fumigatus* isolates from CF patients is pertinent for understanding these dynamics, which is crucial for the patient’s prognosis, preventing the spread of infection within the hospital environment, and minimizing the risk of patient contamination.

As mentioned earlier, the susceptibility profile varies among *A. fumigatus* genotypes [18], and azole resistance can either evolve (clinical resistance) or be selected after azole exposure (environmental resistance) [21,22,47,48,49,50]. The primary mechanism of azole resistance in *A. fumigatus* is attributed to *cyp*51A gene mutations [26]. Five isolates from two patients harbored the N248K point mutation. The N248K alteration, when combined with a V436A point mutation, is associated with azole resistance. Chen et al. [51] demonstrated this combination in vitro, revealing that the artificially constructed strain exhibited higher MICs for azoles, particularly ITC (>16 μg/mL), compared to an isolate with the N248K mutation alone. Although the N248K mutation, when combined with others, may result in elevated MICs for azoles, it can also be present in susceptible isolates, as reported in the literature [52,53].

Two isolates carrying five mutations (F46Y, M172V, N248T, D255E, E427K) in the *cyp*51A gene also tested positive in the surveyor technique. However, these mutations are not linked to azole resistance and have been previously identified in resistant and susceptible isolates [54,55,56]. Snelders et al. [27] examined 76 *A. fumigatus* isolates and found the same five mutations in 13 isolates with low MIC values against azoles and one resistant isolate. Similar mutations have been reported in various studies involving resistant and susceptible isolates [57,58,59]. Consequently, these five amino acid changes are not proximate to any of the *cyp*51A protein domains interacting with antifungal compounds; therefore, no impact on the biological protein activity is anticipated [27]. Nevertheless, monitoring these sequential isolates remains crucial, given the detection of resistant clinical isolates in our institution and other facilities across Brazil [34,49,50,60].

In our analysis, it was noted that two clonal complexes were shared among three CF patients, a phenomenon attributable to potential hospital contamination. Additionally, three patients consistently harbored colonization by the same clonal complex, indicating a degree of stability in their microbial communities. Co-colonization or co-infection involving multiple genotypes was observed across all patients, underscoring the complexity of *A. fumigatus* dynamics within the CF respiratory tract.

Notably, the strains investigated herein demonstrated no discernible azole resistance, despite two patients undergoing azole treatment. However, specific isolates displayed amino acid alterations in *cyp*51A, although these mutations did not confer phenotypic resistance to azole. Therefore, monitoring these patients who were chronically colonized by *A. fumigatus* becomes even more necessary, as these strains may carry mutations in the *cyp*51A gene and lead to possible therapeutic failure.

The development of point mutations in the *cyp5*1A gene due to the unnecessary use of antifungals is not a reality in our institution, as prophylaxis with antifungals is not currently performed for patients with cystic fibrosis. Although not used, the Sankey diagram can benefit from clearly and understandably visualizing the distribution of risk among patients with cystic fibrosis and the prophylaxis strategies applied, facilitating the analysis of the effectiveness and efficiency of preventive interventions against cystic fibrosis invasive aspergillosis [61].

Continuously surveilling sequential isolates from CF patients assumes paramount significance, considering the previously identified resistant isolates within our institutional cohort, which holds potential implications for therapeutic efficacy. Subsequent investigations are imperative to comprehensively elucidate the nuanced role of *A. fumigatus* colonization and infection in CF patients. This focused inquiry within the CF patient population presents a unique opportunity to refine clinical strategies for optimizing pulmonary health outcomes.

Our findings reveal that certain CF patients can be colonized by *A. fumigatus* strains belonging to the same clonal complexes for extended periods. However, we also identified clinical isolates with the potential for cross-colonization among patients, likely attributed to hospital contamination. Notably, two patients undergoing long-term ITC treatment did not develop clinical resistance. One of these patients exhibited clinical isolates with mutations in the *cyp*51A gene, although not showing phenotypic resistance.

## 5. Conclusions

This study significantly enhances our understanding of *A. fumigatus* dynamics in CF patients over time, mainly focusing on colonization patterns. These nuanced insights deepen our knowledge of how *A. fumigatus* impacts disease progression in cystic fibrosis. The findings also contribute to comprehending the complexities surrounding healthcare-associated infections, especially regarding cross-contamination within healthcare facilities. The evidence presented emphasizes the critical need to address the risk of cross-contamination, particularly in settings where patients undergo prolonged azole treatment, making them more susceptible to developing and exchanging resistant strains. By illuminating these dynamics, this research underscores the urgent need for implementing robust infection control measures to mitigate the spread of resistant pathogens and safeguard patient health. Going forward, it is imperative to prioritize surveillance, stringent hygiene protocols, and antimicrobial stewardship to effectively combat the threat of cross-contamination and its associated risks within healthcare settings.

## Figures and Tables

**Table 1:** Results of the seven positive *A. fumigatus* isolates for the surveyor nuclease technique, *cyp*51A gene sequencing, and Minimum Inhibitory Concentration for the azoles Itraconazole, Voriconazole, and Posaconazole.

Strain	Date	Patient	Clinical Material	Surveyor Nuclease	*cyp*51A Gene	MIC (µg/mL)		
						VRC	ITC	POS
LIF 3061	27 September 2017	7	Sputum	+	F46Y, M172V, N248T, D255E and K427E	2	1	0.5
LIF 2509	20 October 2015	30	Sputum	+	N248K	0.5	0.5	0.5
LIF 2543	17 November 2015	31	Sputum	+	N248K	0.5	0.25	0.25
LIF 3105	9 January 2018	31	Sputum	+	N248K	0.5	0.5	0.5
LIF 3261	21 August 2018	31	Sputum	+	N248K	1	1	0.5
LIF 3318	17 December 2018	31	Sputum	+	N248K	1	0.5	0.25
LIF 3302	28 September 2018	41	Sputum	+	F46Y, M172V, N248T, D255E, and K427E	1	1	0.5

+ Positive for surveyor nuclease assay. VRC: Voriconazole; ITC: Itraconazole; POS: Posaconazole.

## Data Availability

The original contributions presented in the study are included in the article, further inquiries can be directed to the corresponding authors.
